# Temperature-dependence of membrane protein–lipid interactions in membranes†

**DOI:** 10.1039/d5cc01576f

**Published:** 2025-06-23

**Authors:** Smriti Kumar, James Downing, Michael Lynn, Lauren Stover, Carter Lantz, David H. Russell, Arthur Laganowsky

**Affiliations:** a Department of Chemistry, Texas A&M University College Station Texas 77843 USA ALaganowsky@chem.tamu.edu

## Abstract

Membrane protein–lipid interactions, like other biomolecular interactions, are often temperature-sensitive. Here, we use variable-temperature electrospray ionization mass spectrometry to investigate the temperature dependence of protein–lipids interactions from membranes. The findings reveal that specific lipid binding, and in some cases metal ion binding, to membrane proteins is significantly enhanced at elevated temperatures.

## Abstract 2

Membrane protein–lipid interactions, like other biomolecular interactions, are often temperature-sensitive.

## Graphical abstract

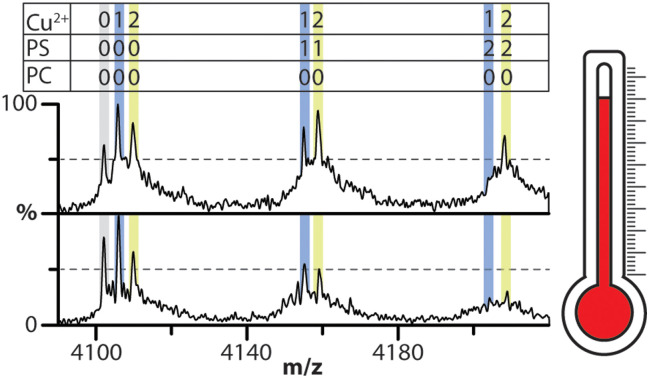

Membrane proteins reside within the chemically diverse lipid environment of the biological membrane, where they engage in non-covalent interactions with surrounding lipids. These interactions are crucial in facilitating specific cellular functions, influencing protein structure and biological activity.^1,2^ The stability and specificity of these molecular interactions are determined by the change in Gibbs free energy, which is the sum of the change in enthalpy and the product of the change in entropy and temperature.^3–5^ The fluidity of the lipid bilayer is influenced by both its composition and temperature. In biological organisms that lack cholesterol, membrane fluidity is regulated by the incorporation of *cis*-unsaturated lipids, which lower the transition temperature, and saturated lipids, which increase the transition temperature.^6^ Earlier electron paramagnetic resonance studies have reported the rate of exchange for lipid–protein interactions is significantly enhanced at elevated temperatures.^7^ Temperature also affects protein activity, such as the bacterial protein DesK, whose kinase activity is activated at cold temperatures.^8^ However, how temperature influences membrane protein**–**lipid interactions in membranes remains poorly understood.

Central to understanding the selectivity and specificity of membrane protein**–**lipid interactions, including their influence on structure and function, is a detailed characterization of their biochemical properties. To this end, native mass spectrometry (MS) is rapidly enabling the detailed characterization of biomolecular interactions.^1,9^ To date, most native MS studies of membrane protein have utilized proteins solubilized in detergent.^10^ This approach has been instrumental in defining the molecular requirements for specific lipid binding to TRAAK, a two-pore domain potassium channel,^11^ and thermodynamic analyses of specific protein**–**lipid interactions.^12,13^ While detergents generally preserve the native structure of membrane proteins in native MS studies,^14,15^ they have also been shown to influence ligand binding in some instances.^16^ Beyond detergent-based methods, the study of membrane proteins within intact bilayers, such as those incorporated in nanodiscs, cell-derived membranes, or liposomes, has demonstrated promise in retaining protein**–**lipid interactions even after ejection from the lipid environment.^17–24^

While equilibrium thermodynamics for membrane protein**–**lipid interactions solubilized in detergent has been well studied,^3,12,25–27^ the temperature dependence of these interactions in a bilayer remains largely unexplored. To address this, we envisioned an approach where a membrane protein is first reconstituted into proteoliposomes with a defined lipid composition (Fig. 1A). These proteoliposomes can then be prepared for native mass spectrometry (MS) analysis and introduced into a variable-temperature electrospray ionization (ESI) apparatus,^3,28^ for MS measurements at different solution temperatures. To analyze membrane protein**–**lipid interactions, proteins bound to lipids can be ejected from proteoliposomes by applying collision energy (Fig. 1B). In this case, the use of a supercharging molecule, such as 3-nitrobenzyl alcohol (m-NBA), has been shown to facilitate the ejection of lipid-bound membrane proteins from nanodiscs and proteoliposomes.^20,24,29^ In parallel, adding detergent to proteoliposomes can solubilize membrane proteins and lipids, providing insights into the role of an intact bilayer (Fig. 1C). Dynamic light scattering (DLS) study shows the size distribution of proteoliposomes and after solubilization with detergent (Fig. 1D). Notably, the addition of a supercharging reagent does not disrupt the size distribution of the proteoliposomes (Fig. S1, ESI†). In both cases (Fig. 1B and C), the temperature dependence of lipid binding can be directly assessed by analyzing the abundance of lipid-bound stoichiometries, offering a unique perspective on membrane protein**–**lipid interactions under different conditions.

**Fig. 1:**
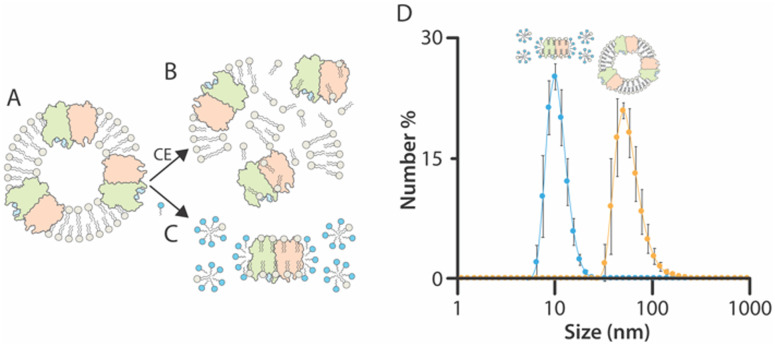
Overview of the native MS approach to study membrane protein–lipid interactions in proteoliposomes. (A) The target protein is incorporated into a proteoliposome of defined composition. (B) Proteoliposomes are introduced into the mass spectrometer, and collision energy (CE) is applied to eject membrane proteins bound to lipids. (C) Detergent is added to the proteoliposomes to solubilize membrane proteins and lipids. (D) Dynamic light scattering of TRAAK in proteoliposomes containing 10% PS and after solubilization with C_10_E_5_. Reported are mean and standard deviations (*n* = 3).

The bacterial trimeric ammonia channel (AmtB) was reconstituted into liposomes of varying compositions. In 1-palmitoyl-2-oleoyl-*glycero*-3-phosphocholine (PC) liposomes, AmtB exhibited no temperature dependence in lipid binding over a range from 25 °C to 37 °C (Fig. 2A and Fig. S2, ESI†). AmtB was then reconstituted into PC liposomes containing 10% or 25% 1,2-dipalmitoyl-*sn-glycero*-3-phosphoethanolamine (PE). While no direct PE binding was observed, the overall abundance of PC-bound stoichiometries increased compared to pure PC proteoliposomes (Fig. 2B and Fig. S3A, S3C, and S4A, ESI†). However, lipid-bound states remained temperature-independent. Similarly, in proteoliposomes containing 1,2-dipalmitoyl-*sn-glycero*-3-phospho-(1′-*rac*-glycerol) (PG), no PG binding was detected, and lipid binding did not vary with temperature (Fig. S5A, S5C, S6A, and S6C, ESI†). In contrast, 1′,3′-bis[1,2-dipalmitoyl-*sn-glycero*-3-phospho]-glycerol (CL) in proteoliposomes revealed a striking temperature dependence in lipid binding profiles. As the temperature increased from 25 °C to 37 °C, the binding of AmtB to PC and mixed lipid stoichiometries increased (Fig. 2C and Fig. S7A, S7C, and S8A, ESI†). Notably, AmtB bound to two PC and two CL molecules was observed only at 37 °C. However, the intensity of AmtB bound to CL exhibited only modest changes with temperature. These findings demonstrate that AmtB–lipid interactions exhibit temperature dependence in a bilayer, particularly in the presence of CL-containing membranes.

**Fig. 2:**
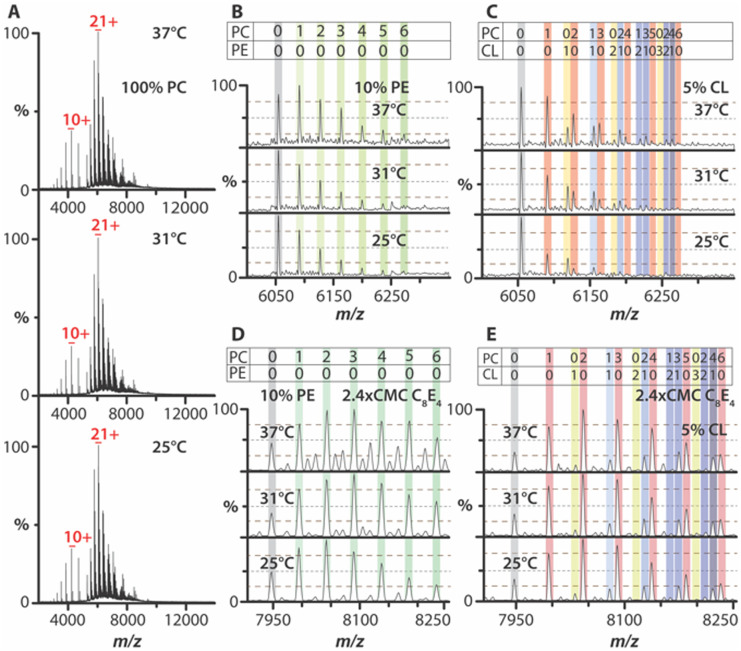
Temperature-dependence of AmtB–lipid interactions in proteoliposomes. (A) Native mass spectra of AmtB in PC liposomes acquired at different solution temperatures. (B) and (C) Mass spectra for the 21^+^ charge state of AmtB ejected from (B) 10% PE liposomes and (C) those containing 5% CL. (D) and (E) Mass spectra for the 16^+^ charge state of AmtB ejected from (D) 10% PE liposomes and (E) those containing 5% CL after solubilizing with C_8_E_4_.

Next, we investigated whether the temperature dependence of AmtB–lipid interactions observed in proteoliposomes would be preserved in detergent-solubilized samples. C_8_E_4_ detergent was selected to disrupt the proteoliposome, as AmtB has been extensively studied in this detergent.^14,15,30^ Detergent-solubilized proteoliposomes (DSPs) containing only PC exhibited no temperature dependence in lipid binding (Fig. S9, ESI†). In contrast, DSPs containing PE displayed a modest temperature dependence, with 25% PE showing a more pronounced increase in lipid binding (Fig. 2D and Fig. S3B, S3D, and S4B, ESI†). PG-containing DSPs showed no temperature dependence, consistent with observations from intact proteoliposomes (Fig. S5 and S6, ESI†). Interestingly, 5% CL-containing DSPs lost the temperature-dependent lipid binding observed from intact proteoliposomes (Fig. 2E and Fig. S7B, ESI†). While 10% CL-containing DSPs exhibited some temperature dependence, with increased intensity for certain PC and mixed lipid-bound states, the effect was less pronounced than from intact proteoliposomes (Fig. S7D and S8B, ESI†). These findings suggest that temperature-dependent AmtB–lipid interactions are significantly altered after detergent solubilization, highlighting a difference between intact bilayers and detergent-solubilized environments.

Next, we investigated the human TWIK-related arachidonic acid-stimulated potassium channel (TRAAK), a two-pore domain potassium channel involved in maintaining the resting membrane potential and other physiological processes.^31^ Previous studies have shown that TRAAK selectively binds 1-palmitoyl-2-oleoyl-*sn-glycero*-3-phosphate (PA) and that Cu^2+^ can selectively modulate TRAAK-PS interactions.^31,32^ Inspired by these findings, TRAAK was reconstituted into PC liposomes containing 10% PA (Fig. 3A and Fig. S10A, ESI†). PA-containing TRAAK proteoliposomes displayed broad mass distributions, occasionally accompanied by sharp mass spectral peaks corresponding to distinct Cu^2+^ bound states (Fig. 3A). Notably, the first and second Cu^2+^ binding exhibited a strong temperature dependence, with enhanced binding at higher temperatures. Upon addition of diethylenetriaminepentaacetic acid (DTPA), a high-affinity Cu^2+^ chelator, both metal ion binding and lipid-bound states were abolished (Fig. 3B and Fig. S10B, ESI†).

**Fig. 3:**
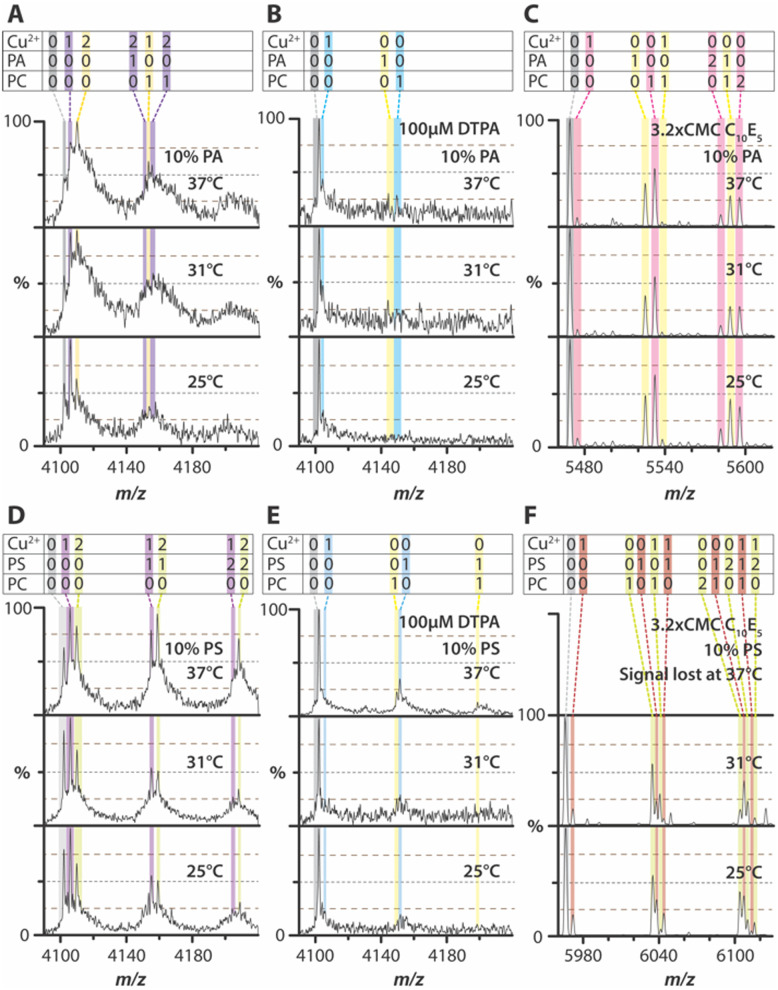
Temperature-dependence of TRAAK-lipid interactions in proteoliposomes. (A)–(C) Zoom of the mass spectra of TRAAK in proteoliposomes consisting of (A) 10% PA, (B) 10% PA with 100 μM DTPA, and (C) 10% PA with 3.2 × CMC C_10_E_5_. (D)–(F) Zoom of the mass spectra of TRAAK in proteoliposomes consisting of (A) 10% PS, (B) 10% PS with 100 μM DTPA, and (C) 10% PS with 3.2 × CMC C_10_E_5_. For proteoliposomes, the 16^+^ charge state is shown, whereas for detergent-solubilized samples, the 11^+^ charge state is shown.

To further investigate Cu^2+^ binding, TRAAK was reconstituted into 1-stearoyl-2-oleoyl-*sn-glycero*-3-phospho-l-serine (PS)-containing liposomes (Fig. 3D and Fig. S11A, ESI†). The resulting mass spectra revealed well-defined peaks for different Cu^2+^ binding stoichiometries, which were more pronounced than PA-containing proteoliposomes. Temperature had a striking effect on the abundance of metal ion-bound states. While TRAAK binding to two Cu^2+^ increased at elevated temperatures, the presence of PS led to a dramatic enhancement of two Cu^2+^ binding at 37 °C. The addition of DTPA eliminated both metal ion-bound states and TRAAK–lipid complexes (Fig. 3E and Fig. S11B, ESI†). These findings demonstrate that Cu^2+^ binding to TRAAK in a bilayer is enhanced at 37 °C, with an even more significant effect observed in the presence of PS.

To better understand the impact of lipids on Cu^2+^ binding to TRAAK, we also reconstituted TRAAK into PC and 9 : 1 PC : PG proteoliposomes (Fig. S12A and S13A, ESI†). The Cu^2+^ binding profiles are markedly different from those observed in PA- or PS-containing proteoliposomes (Fig. S14A and S15A, ESI†). Unlike in PA- or PS-containing liposomes, the binding of two Cu^2+^ was not detected, though a moderate signal for a single Cu^2+^ was observed. Additionally, Cu^2+^ binding was not influenced by temperature. Upon the addition of DTPA, the mass spectral peaks improved (Fig. S12B, S13B, S14B and S15B, ESI†). Interestingly, a peak corresponding to the mass of a potassium ion binding to the channel was detected, but it did not exhibit any temperature dependence.

To further investigate Cu^2+^ binding, similar experiments were conducted for AmtB by solubilizing TRAAK proteoliposomes with C_10_E_5_, a detergent previously used for native MS studies of the channel.^31,32^ In general, DSPs exhibited a reduction in Cu^2+^ binding, and neither Cu^2+^ nor lipid binding showed temperature dependence (Fig. 3C, F and Fig. S10C, S11C, S12C, S13C, S14C, and S15C, ESI†). In some cases, no mass spectrum could be acquired at the highest temperature investigated, suggesting that the intact bilayer plays a crucial role in maintaining thermal stability and/or preventing aggregation of the channel. Furthermore, the addition of DTPA effectively disrupted Cu^2+^ binding, particularly in PS-containing DSPs (Fig. S10D, S11D, S12D, S13D, S14D, S15D, S16, ESI†). These findings indicate that Cu^2+^ binding to TRAAK depends not only on lipid composition but is also enhanced in the presence of a bilayer, emphasizing the critical role of the membrane environment in modulating metal ion interactions.

These studies reveal the temperature-dependent nature of membrane protein**–**lipid interactions in membranes, offering valuable insights into the molecular recognition of these non-covalent interactions. In these experiments, the only variable altered is the solution temperature. All other conditions, including instrument settings for a given sample, are held constant. Therefore, any observed changes in the mass spectra can be directly attributed to temperature-dependent shifts in solution-phase equilibria. While some protein**–**lipid interactions exhibit temperature sensitivity, others remain unaffected. In all cases, PC serves as the bulk lipid component, yet other lipids can be preferentially bound despite the (*e.g.*, 9-fold molar) excess of PC. For example, in AmtB, the binding of mixed CL and PC states is enhanced at elevated temperatures. In TRAAK, Cu^2+^ binding to the channel is influenced by both an intact bilayer (containing PS or PA) and temperature, as evidenced by the selective increase in the binding of PS and Cu^2+^. These findings highlight that temperature significantly modulates membrane protein**–**lipid interactions within an intact bilayer.

Detergent solubilization of proteoliposomes disrupted the temperature dependence of lipid and metal ions binding to membrane proteins in an intact bilayer. This contrasts with previous studies on the thermodynamics of membrane protein**–**lipid interactions in detergent, where lipid binding, in some cases, exhibited significant temperature dependence.^25,26,30^ For example, the specific binding of phosphoinositides to the inward rectifier potassium channel Kir3.2 was shown to be driven by either enthalpy or entropy, depending on conditions.^26^ However, it is important to note that these previous equilibrium thermodynamic studies were conducted using relatively low lipid concentrations, typically involving a single lipid type added to purified membrane proteins. This approach differs significantly from the present study, where the membrane lipid-to-protein ratio is approximately 1000 : 1, and DSPs contain a mixed lipid environment. Given these differences, further studies are needed to better characterize the temperature dependence of membrane protein**–**lipid interactions, both in proteoliposomes and detergent-solubilized systems. Such investigations will offer deeper insights into how membrane environments influence lipid binding dynamics under different conditions.

In summary, the temperature dependence of lipid binding in a bilayer provides information about the binding thermodynamics. For AmtB, CL binding is enhanced at elevated temperatures consistent with an entropy-driven process. For TRAAK, Cu^2+^ and PS binding also follow an entropy-driven process with enhanced binding at elevated temperatures. In both cases, a decrease in binding at a higher temperature is not observed, as expected for a process with an unfavorable entropy associated. Some protein**–**lipid interactions are unaffected by temperature, consistent with an enthalpically driven reaction. So, what might contribute to entropy-driven protein**–**lipid interactions in a bilayer? A contributing factor is the desolvation of the lipid headgroup, metal ions, and specific binding sites at a higher temperature, where removed solvent molecules are transferred to the bulk, thereby increasing the number of degrees of freedom and favoring the binding with the protein.^12,26,33^ Another consideration is the rearrangement of the molecular interactions between lipids (*e.g.*, hydrogen bonds between headgroups)^34^ and those newly formed with the protein. While lipid dynamics generally increase with temperature, only a subset exhibit temperature-dependent protein binding, suggesting selective lipid–protein interactions influenced by specific structural and energetic factors. However, further studies are needed to investigate how cholesterol and acyl chain chemistry influence the temperature dependence of protein**–**lipid interactions.

This work was supported by Welch Foundation (A-2106-20220331) and NIH (R01GM139876 and RM1GM1454316) awarded to A. L., and NIH (RM1GM149374) awarded to D. R.

## Conflicts of interest

The authors declare no conflicts of interest.

## Supplementary Material

CC-061-D5CC01576F-s001

## Data Availability

The data supporting this article have been included as part of the ESI.†
