# Supplementary material for: Temperature-dependence of membrane protein–lipid interactions in membranes
Source: Chem Commun (Camb). 2025 Jun 23;61(61):11429–32. doi: 10.1039/d5cc01576f (PMC12204147; doi:10.1039/d5cc01576f)
Supplement: CC-061-D5CC01576F-s001 [file CC-061-D5CC01576F-s001.pdf]

Supplementary Information (SI) for ChemComm.
This journal is © The Royal Society of Chemistry 2025

Supporting Information

Temperature-Dependence of Membrane Protein-Lipid Interactions in an Intact Bilayer

Smriti Kumar,[1] James Downing,[1] Michael Lynn,[1] Lauren Stover,[1] Carter Lantz,[1] David H. Russell,[1] and Arthur Laganowsky[1*]

[1]Department of Chemistry, Texas A&M University, College Station, Texas 77843, United States

*Correspondence: ALaganowsky@chem.tamu.edu

Methods

AmtB Expression and Purification. The AmtB from Ecoli was expressed with a HRV3C protease cleavable N-terminal His_8 tag from pCDF in *E. coli* BL21-A1 (Invitrogen and purified as previously described.[1, 2] Multiple colonies were used to inoculate LB Miller (IBI Scientific) and grown overnight at 37°C. The overnight culture was used to inoculate terrific broth (TB, IBI Scientific) and allowed to grow at 37°C until it reached an OD_{600} of 0.6. Protein expression was induced with the addition of arabinose to a final concentration of 0.2% and grown for 18 h at 20°C. Cells were harvested by centrifugation at 5,000 xg for 10 min, resuspended in lysis buffer (150 mM sodium chloride, 50 mM Tris; pH 7.4 at room temperature), and lysed using a Microfluidics M-110P microfluidizer operating at 25,000 PSI. The lysate was clarified through centrifugation (25 min at 20,000 xg at 4°C). The supernatant was centrifuged (2 h at 100,000 xg at 4°C) to pellet the membrane. The membrane was resuspended in a buffer (100 mM sodium chloride, 20 mM Tris, 20% glycerol; pH 7.4 at room temperature) and extracted overnight with 5% octyl glucoside (OG) at 4°C. The extracted protein mixture was centrifuged at 20,000 xg for 25 mins. The supernatant was filtered with a 0.45-micron syringe filter (Pall Corp.) and loaded onto a HisTrap HP 5 mL column (Cytiva) pre-equilibrated with NHA-DDM buffer (200 mM sodium chloride, 20 mM Tris, 20 mM imidazole, 10% glycerol, 0.025% DDM; pH 7.4 at room temperature). AmtB was eluted with NHB-DDM buffer (100 mM sodium chloride, 20 mM Tris, 500 mM imidazole, 10% glycerol, 0.025% DDM; pH 7.4 at room temperature). The eluted protein was then loaded onto a HiPrep™ 26/10 Desalting column (Cytiva) equilibrated with the NHA-DDM buffer. The protein was pooled, HRV3C was added in a 50:1 protein-to-protease ratio, and the mixture was incubated overnight at 4°C. The cleaved protein was loaded onto a HisTrap HP column equilibrated in NHA-DDM buffer, and the flow-through containing the tag-less protein was collected and concentrated using a 100 kDa MWCO concentrator (MilliporeSigma). Concentrated protein was loaded onto a Superdex 200 Increase 10/300 GL column (GE Healthcare) equilibrated with GF buffer (100 mM sodium chloride, 20 mM Tris, 10% glycerol, 0.5% C_8E_4; pH 7.4 at room temperature). The peak fractions containing C_8E_4-solubilized AmtB were pooled, aliquoted into 50 µL shots, flash-frozen in liquid nitrogen, and stored at -80°C.

TRAAK Expression and Purification. TRAAK (K2p4.1b, Addgene #158744) was expressed and purified as previously described.[3] Glycerol stock containing *Komagataella phaffii* SuperMan5 (och1-Δ1, GAP-mannosidaseHDEL, pep4-Δ1, prb1-Δ1) strain (BioGrammatics Inc.) with the TRAAK plasmid was added to 50 mL YPD (1% yeast extract, 2% tryptone, 2% dextrose) and was grown overnight at 30 °C. This overnight culture was used to inoculate 600 mL of BMGY and was grown overnight at 30 °C. The cells were pelleted at 2000 xg and were media-exchanged into 600 mL BMMY (0.5% methanol). Anti-foaming agent polypropylene glycol 2000 was added to a final concentration of 0.01% and was shaken for 48 h at 27 °C. The cells were harvested by centrifugation at 2000 xg for 5 mins and resuspended into the lysis buffer (150 mM KCl, 50 mM tris, pH 7.4 at room temperature). They were lysed with a Microfluidics M-110P microfluidizer operating at 30,000 PSI. The lysate was clarified through centrifugation (25 min at 20,000 xg at 4°C). The supernatant was centrifuged (2 h at 100,000 xg at 4°C) to pellet the membrane. The membrane was resuspended in a buffer (150 mM KCl, 50 mM Tris, 20% glycerol; pH 7.8 at room temperature) and extracted overnight with 2% DDM at 4°C. The extracted protein mixture was centrifuged at 20,000 xg for 25 mins. The supernatant was filtered with a 0.45-micron syringe filter (Pall Corp.) and loaded onto a HisTrap HP 5 mL column (Cytiva) pre-equilibrated with KHA-DM buffer (150 mM KCl, 50 mM Tris, 20 mM imidazole, 10% glycerol, 0.2% DM; pH 7.8 at room temperature). TRAAK was eluted with KHB-DM buffer (150 mM KCl, 50 mM Tris, 500 mM imidazole, 10% glycerol, 0.2% DM; pH 7.8 at room temperature). Eluted protein was loaded onto a drip column containing Strep-Tactin Sepharose resin (4 mL bed volume, prepared in-house, iba) equilibrated in SPKHA-DM buffer (150 mM KCl, 50 mM Tris, 10% glycerol, 0.2% DM; pH 7.8 at room temperature) and was eluted with SPKHA-DM supplemented with 3 mM desthiobiotin. The eluted protein was then loaded onto a HiPrep™ 26/10 Desalting column (Cytiva) equilibrated with the KHA-DM buffer. TEV protease was added at a 20:1 protein-to-protease ratio, and the mixture was incubated overnight at 4°C. The cleaved protein was loaded onto a drip column containing Ni-NTA superflow (4 mL bed volume, prepared in-house, QIAGEN) equilibrated in KHA-DM buffer. The flow-through was collected and loaded onto a drip column containing Strep-Tactin Sepharose resin (4 mL bed volume, prepared in-house, IBA bioscience) equilibrated in SPKHA-DM buffer. This second flow-through was concentrated using a 50 kDa MWCO

concentrator (MilliporeSigma). Concentrated protein was loaded onto a Superdex 200 Increase 10/300 GL column (GE Healthcare) equilibrated with SPKHA-DM. The peak fractions containing TRAAK were pooled, aliquoted into 50 µL shots, flash-frozen in liquid nitrogen, and stored at -80°C.

Preparation of proteoliposomes for native mass spectrometry analysis. Proteoliposomes were prepared as previously described with minor modifications.[3] In brief, lipids were aliquoted and dried under nitrogen flow and kept under vacuum overnight to remove any trace of organic solvent. The lipid film was hydrated with water to a final concentration of 20 mM. This lipid stock was diluted to 10 mM with appropriate buffer (final concentration of 20 mM HEPES, 150 mM KCl, or NaCl at pH 7.4 for TRAAK and AmtB, respectively). After dilution, the lipid was extruded using a 100 nm membrane filter (Cytiva) until the solution turned translucent. DM was added to a final 5-10x critical micelle concentration (CMC) to solubilize the mixture and was rotated at 4°C for 1 h. membrane protein was added to the solubilized lipid at a protein-to-lipid molar ratio of 1:750, and the mixture was rotated at 4°C for 1 h. In the meantime, bio-beads (Bio-Rad) were prepared by washing them with methanol (1 time), water (5 times), and buffer (3 times). Biobeads were then added to the protein-lipid mixture and rotated at room temperature for 2 hr to remove detergents. The proteoliposome mixture was removed from the biobeads and then extruded using a 100 nm membrane filter (Cytiva). The samples were then dialyzed using a 20 kDa MWCO dialysis membrane overnight against 1L of 200 mM ammonium acetate (AA) at pH 7.4 at 4°C. Before introducing the proteoliposome into the mass spectrometer, supercharging agent m-NBA (3-Nitrobenzyl alcohol, Ambeed Chemicals) was mixed to a final concentration of 0.5% to facilitate the ejection of membrane protein complexes from the liposome.[4] For the detergent-solubilized samples, C_8E_4 or $C_{10}E_5$ was added to the proteoliposome to a final concentration of 2.4x CMC and 3.2x CMC, respectively. The protein concentration in the detergent-solubilized samples was adjusted to a final concentration of 1 µM. For chelating Cu^{2+} from proteoliposomes containing TRAAK, DTPA was added to either proteoliposome or detergent-solubilized samples to the final concentration of 100 µM before introducing it into the mass spectrometer. m-NBA was not required for the detergent-solubilized samples.

Native Mass Spectrometry (MS). Data were collected on an Exactive Plus EMR Orbitrap Mass Spectrometer (Thermo Scientific) modified with a variable temperature apparatus.[5] Samples were loaded into gold-coated borosilicate nanoelectrospray ionization emitters prepared in-house.[6] Detailed instrument settings can be found in Tables S1-4. MS data were analyzed using UniDec.[7]

Dynamic Light Scattering (DLS). DLS experiments were performed on a Zetasizer Nano ZS by Malvern Panalytical. Proteoliposomes with or without m-NBA and detergent-solubilized proteoliposomes were used in the same condition as in the native MS. For AmtB in C_8E_4 (Figure S1), a 50 µL AmtB shot was buffer-exchanged into the mass spectrometry buffer (200 mM AA, 0.5% C_8E_4; pH 7.4) with a centrifugal desalting column (Micro Bio-Spin 6 Columns, Bio-Rad) and diluted further with the same mass spectrometry buffer to a final concentration of 1 µM.

Supporting Figures

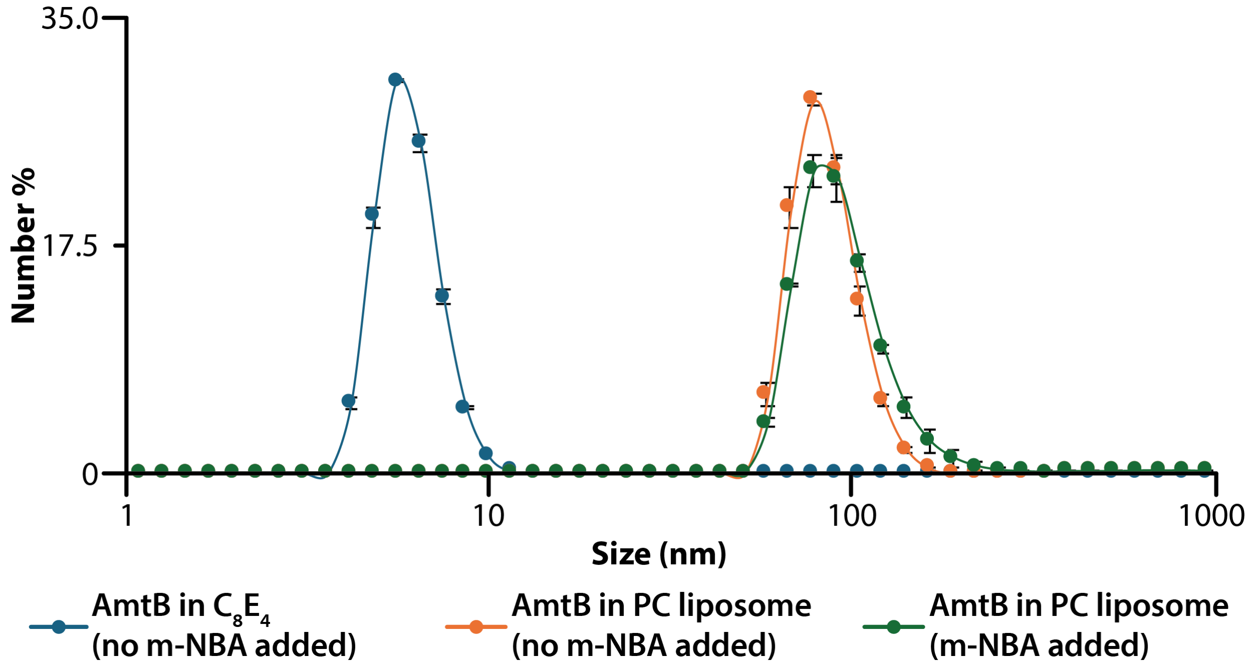

Figure S1. Dynamic light scattering of AmtB in proteoliposomes and detergent. Shown is AmtB in C_8E_4 prior to reconstitution into PC liposomes. The addition of m-NBA, supercharging agent to facilitate ejection of lipid bound membrane proteins from proteoliposomes, does not perturb the size distribution. Reported are mean and standard deviations (n=2).

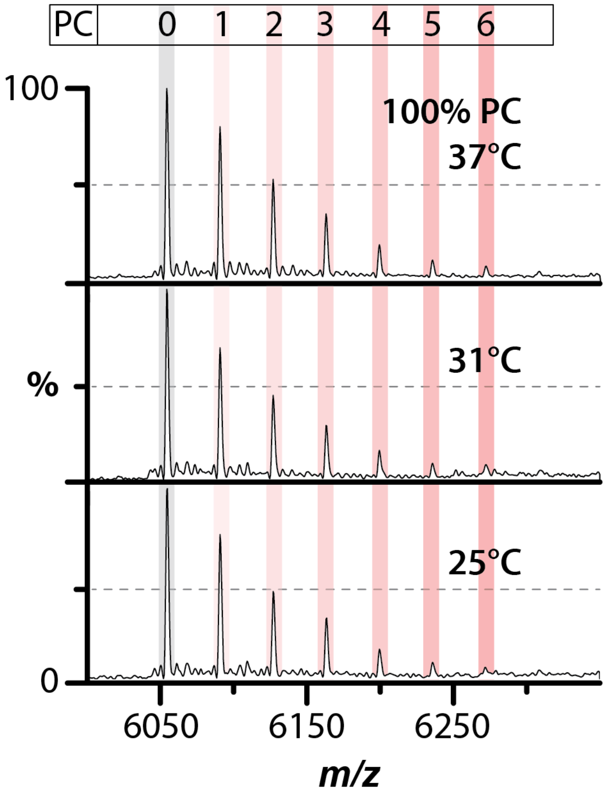

Figure S2. AmtB reconstituted into PC liposomes. 21^+ charge state has been zoomed.

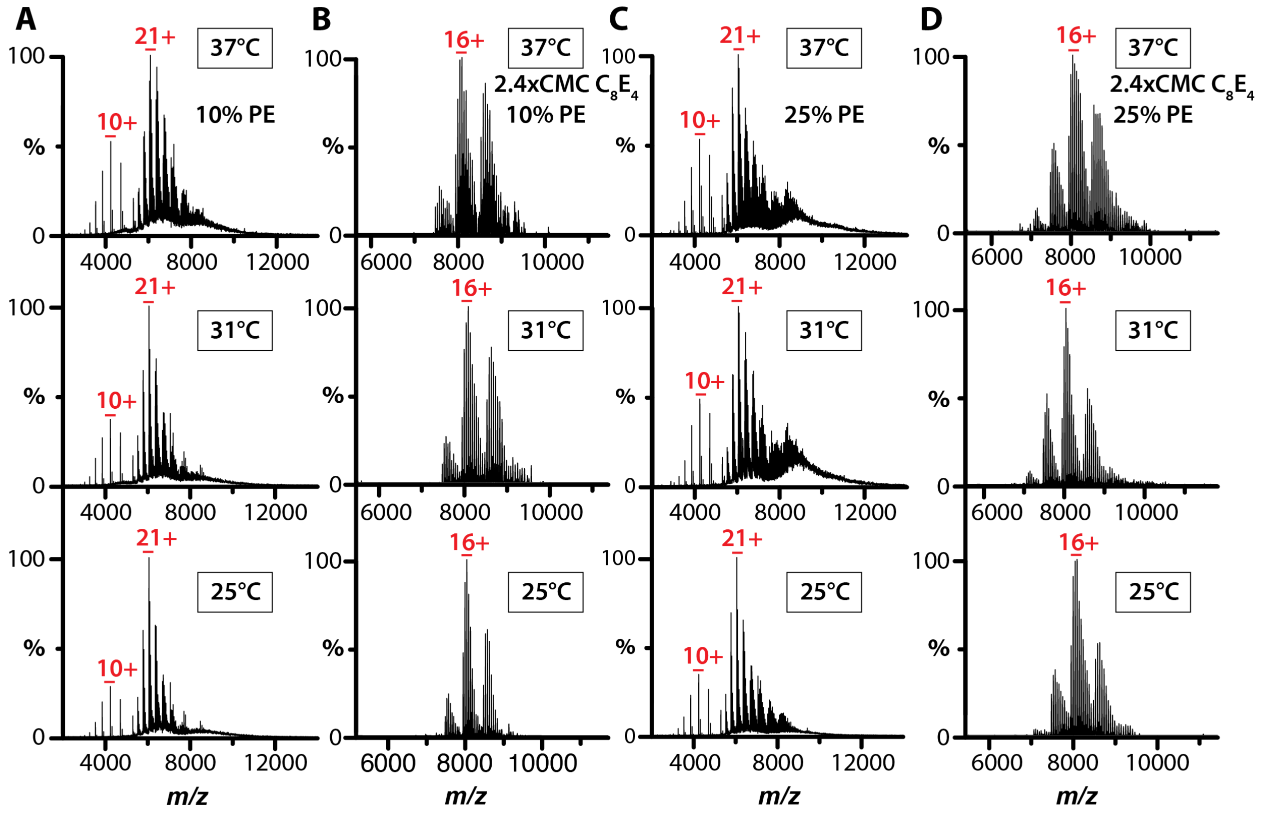

Figure S3. AmtB in proteoliposomes containing different percentages of PE. Native mass spectra of AmtB in proteoliposomes consisting of A) 10% PE, B) 10% PE solubilized with C_8E_4, C) 25% PE, and D) 25% PE solubilized with C_8E_4. The solution temperature is denoted in the inset.

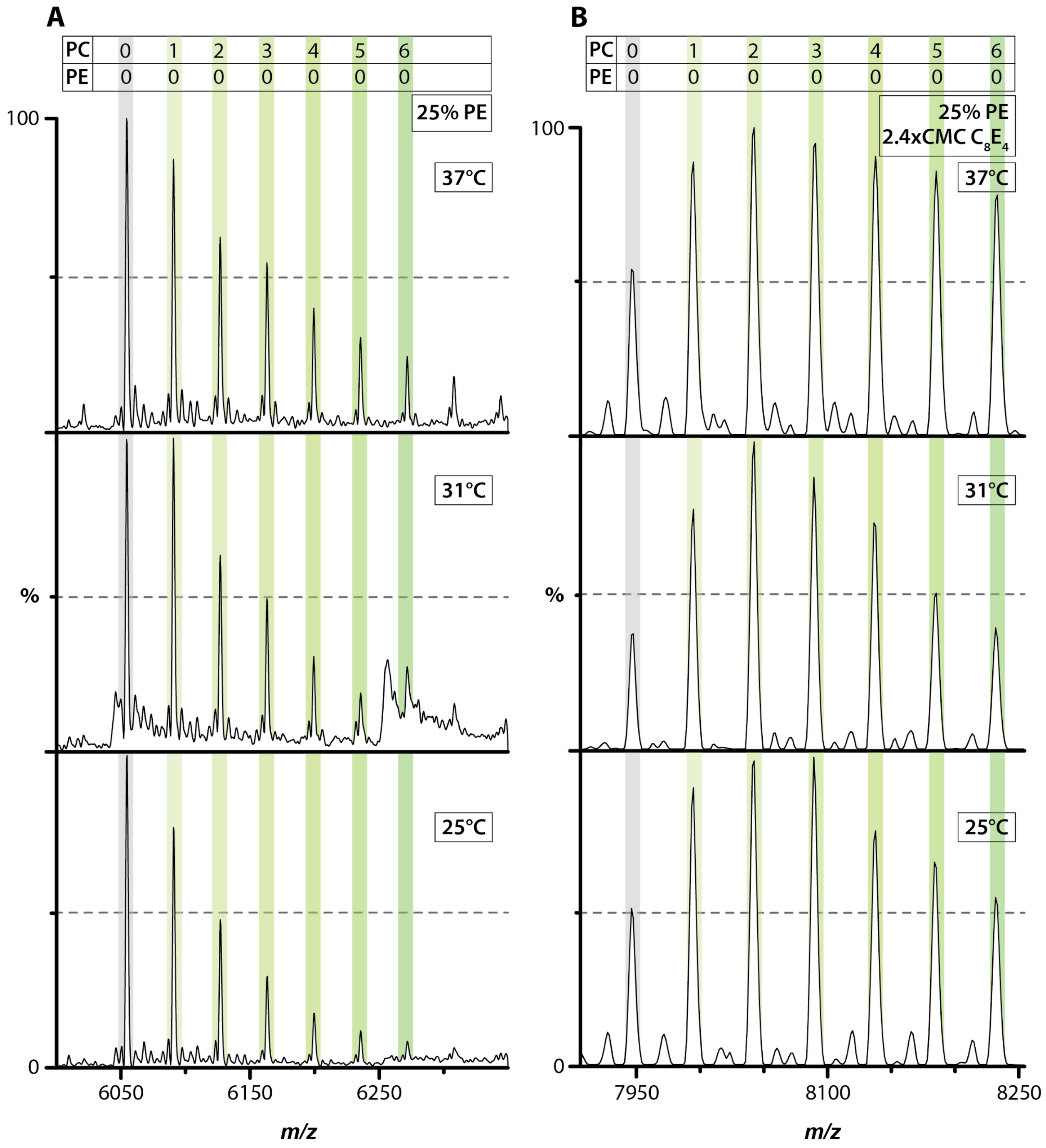

Figure S4. AmtB in proteoliposomes containing different percentages of PE. A) Zoom of 21+ charge state from the mass spectra of AmtB in proteoliposome consisting of 25% PE, B) Zoom of 16+ charge state from the mass spectra of AmtB in proteoliposome consisting of 25% PE solubilized with C_8E_4.

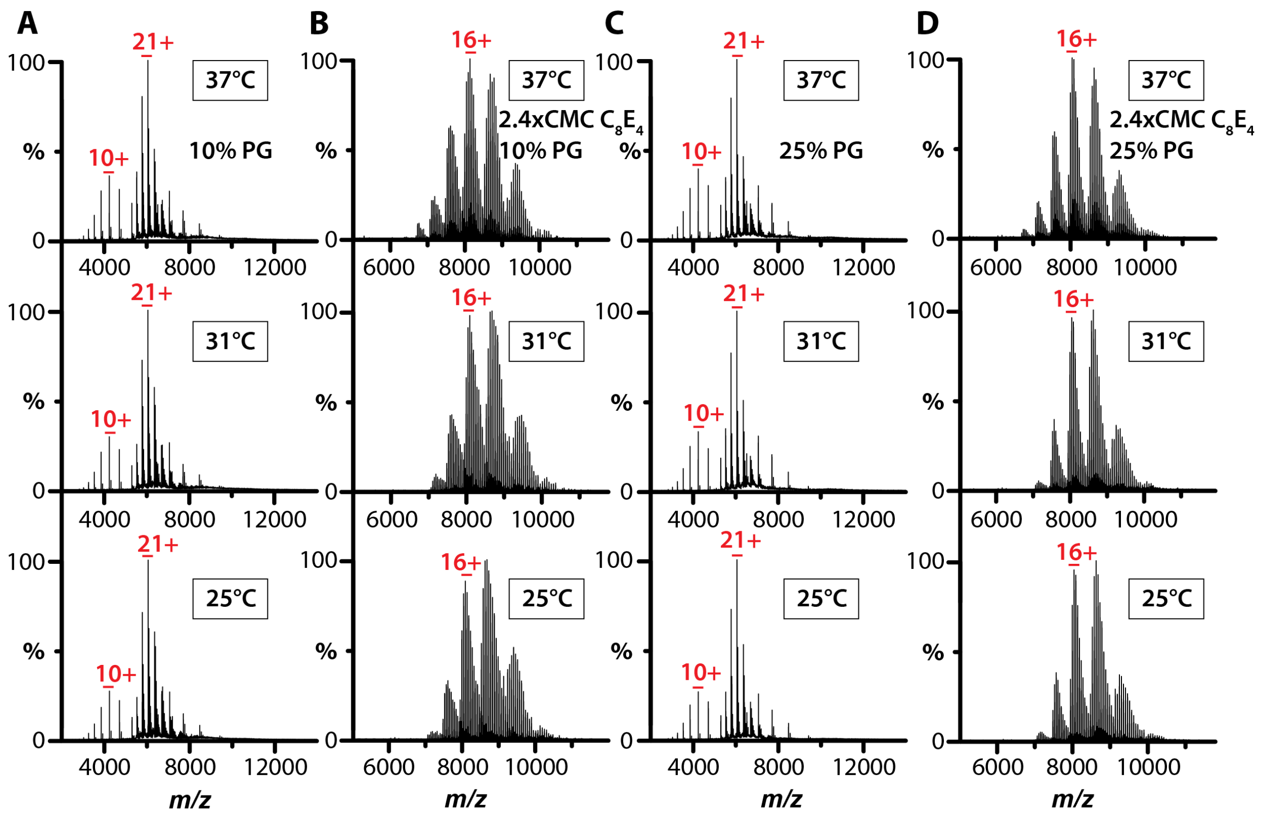

Figure S5. AmtB in proteoliposomes containing different percentages of PG. Native mass spectra of AmtB in proteoliposomes consisting of A) 10% PG, B) 10% PG solubilized with C_8E_4, C) 25% PG, and D) 25% PG solubilized with C_8E_4. The solution temperature is denoted in the inset.

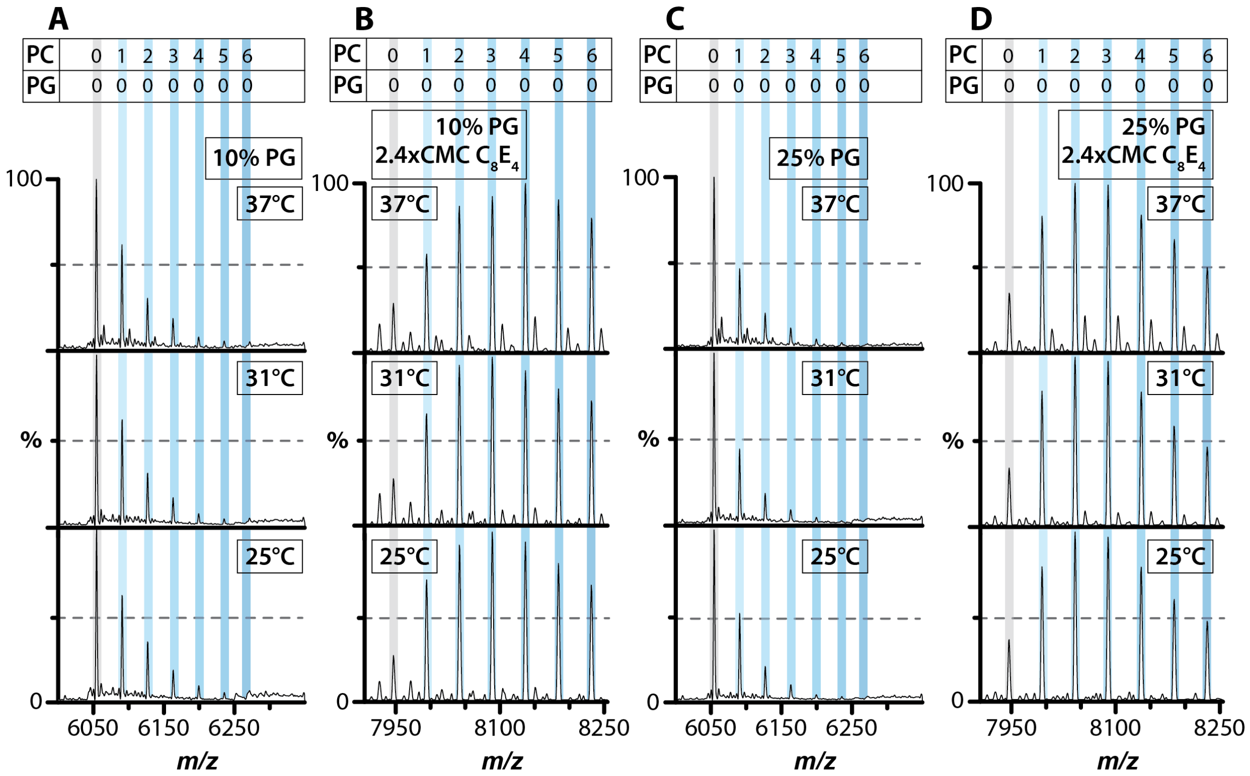

Figure S6. AmtB in proteoliposomes containing different percentages of PG. Zoom of a charge state from the mass spectra of AmtB in proteoliposomes consisting of A) 10% PG, B) 10% PG solubilized with C_8E_4, C) 25% PG, and D) 25% PG solubilized with C_8E_4. For intact proteoliposomes with AmtB, charge state 21+ was zoomed, whereas for detergent-solubilized proteoliposomes, charge state 16+ was zoomed.

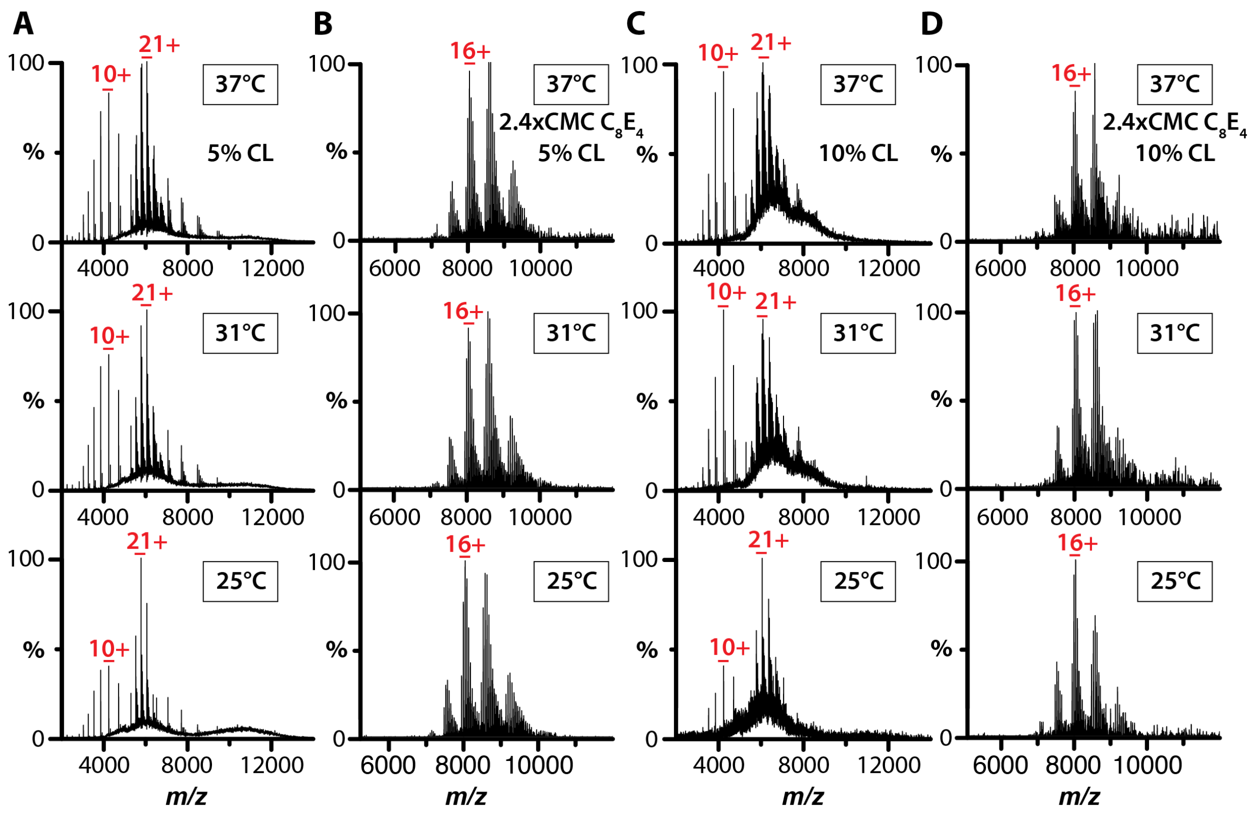

Figure S7. AmtB in proteoliposomes containing different percentages of CL. Native mass spectra of AmtB in proteoliposomes consisting of A) 5% CL, B) 5% CL solubilized with C_8E_4, C) 10% CL, and D) 10% CL solubilized with C_8E_4.

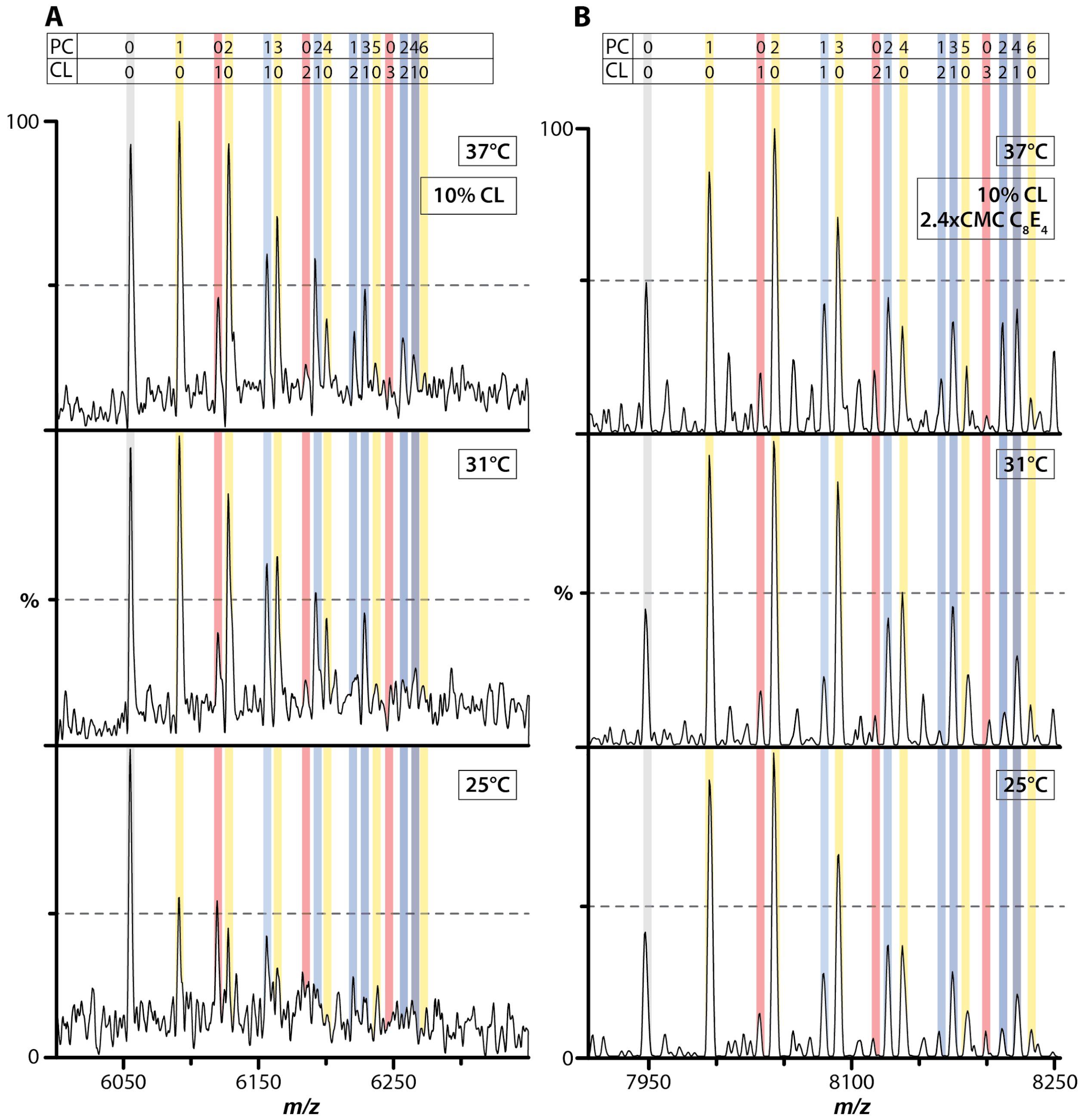

Figure S8. AmtB in proteoliposome containing 10% CL. A) Zoom of 21+ charge state from the mass spectra of AmtB in proteoliposome consisting of 10% CL, B) Zoom of 16+ charge state from the mass spectra of AmtB in proteoliposome consisting of 10% CL solubilized with C_8E_4.

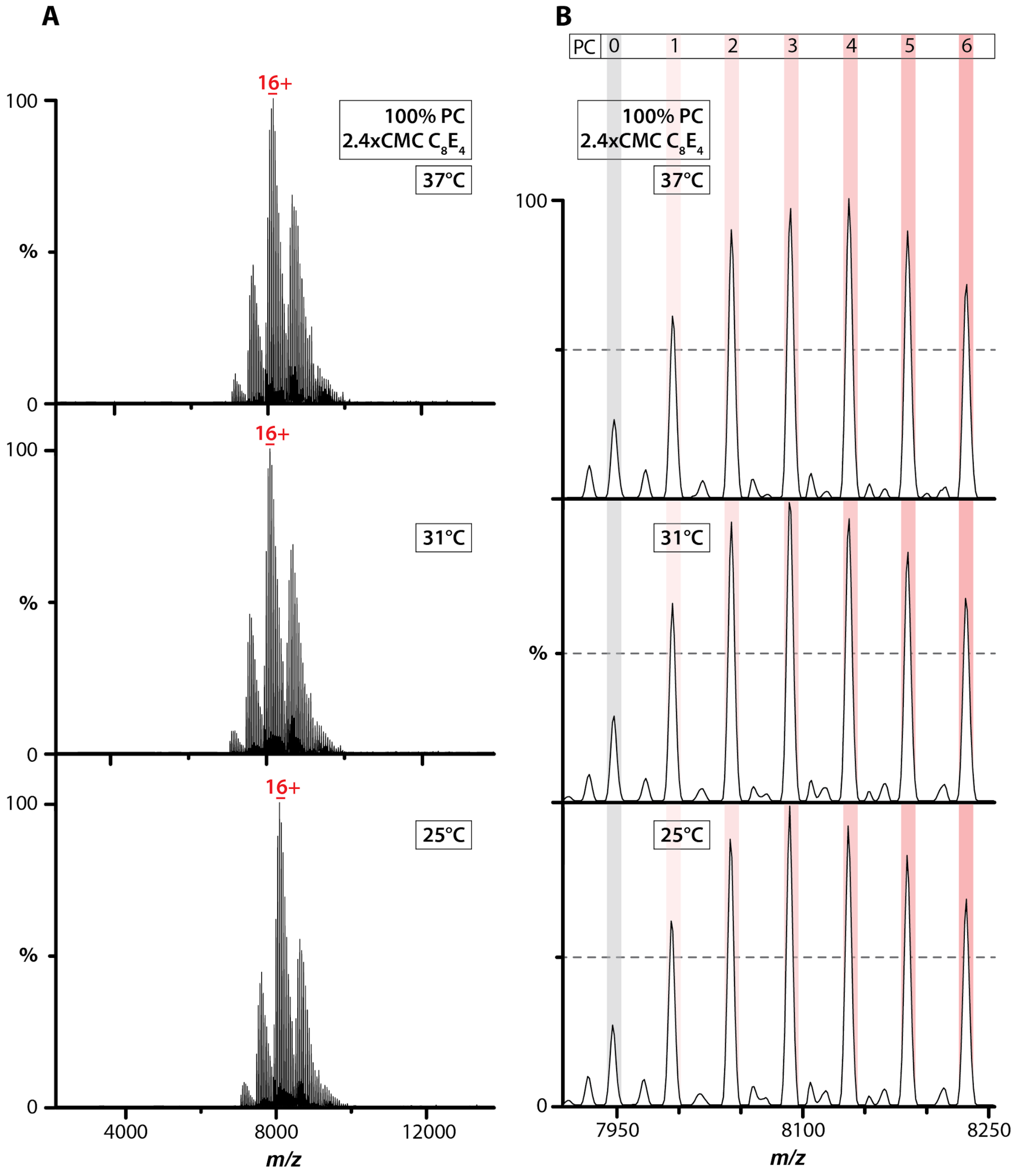

Figure S9. AmtB in proteoliposome recorded at various temperatures. A) Native mass spectra AmtB in 100% POPC, solubilized with C_8E_4. B) Zoom of 16+ charge state of the same spectra.

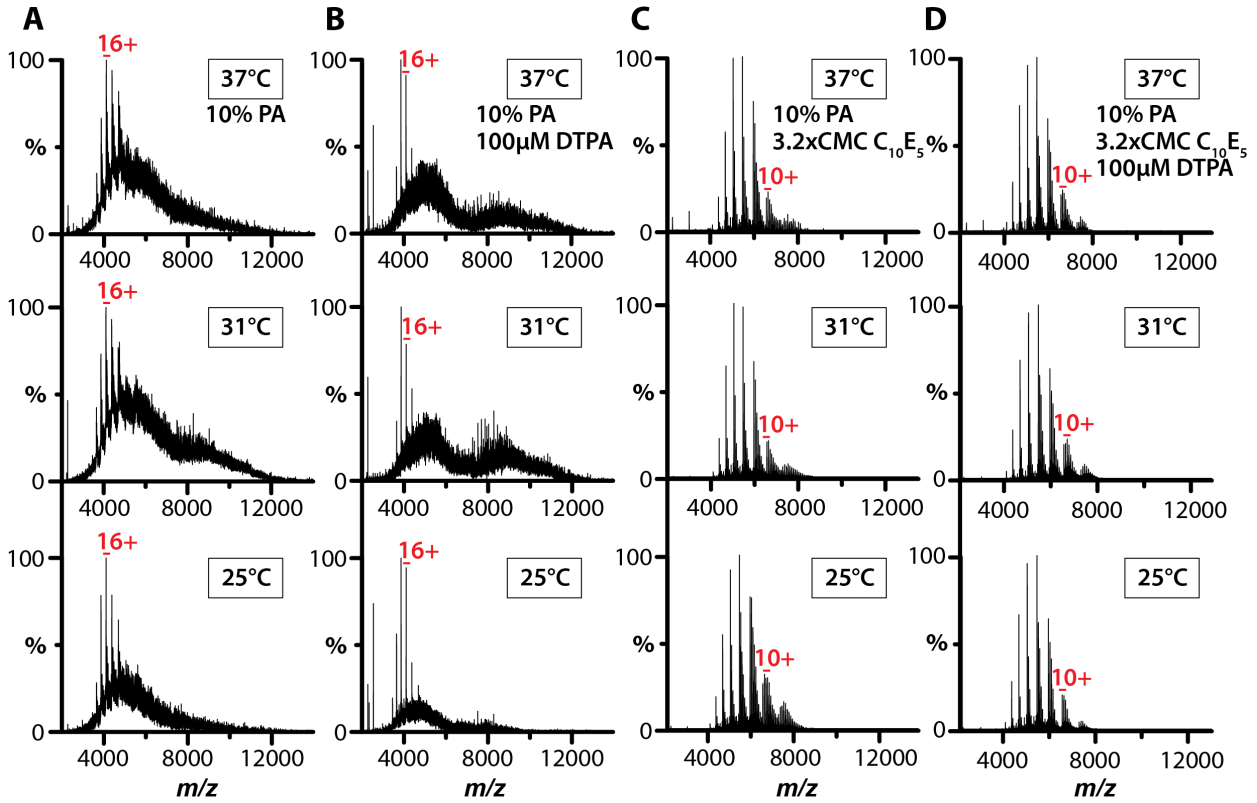

Figure S10. TRAAK in proteoliposomes containing PA recorded at different temperatures. Native mass spectra of TRAAK consisting of A) 10% PA, B) 10% PA with 100µM DTPA, C) 10% PA with 3.2xCMC $C_{10}E_5$, and D) 10% PA with 100µM DTPA, and 3.2xCMC $C_{10}E_5$.

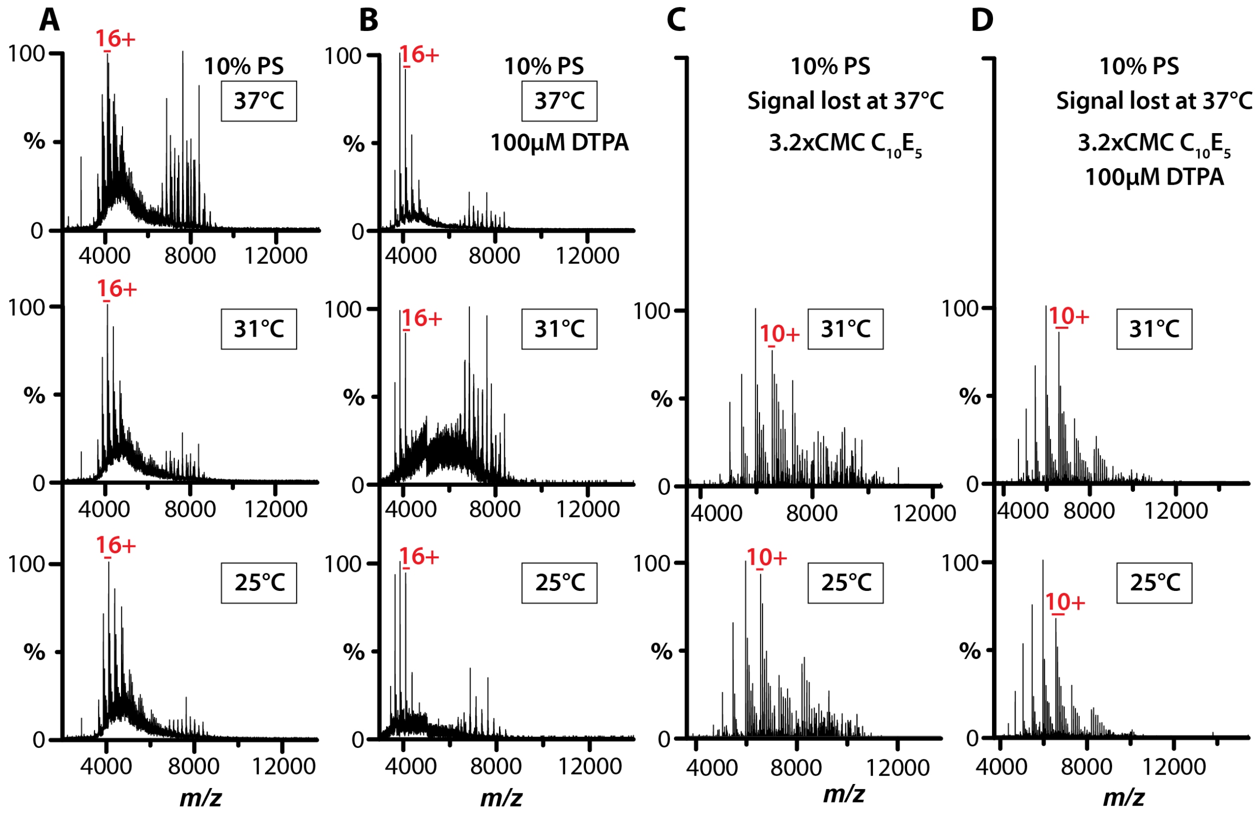

Figure S11. TRAAK in proteoliposomes containing PS recorded at different temperatures. Native mass spectra of TRAAK consisting of A) 10% PS, B) 10% PS with 100µM DTPA, C) 10% PS with 3.2xCMC $C_{10}E_5$, and D) 10% PS with 100µM DTPA, and 3.2xCMC $C_{10}E_5$.

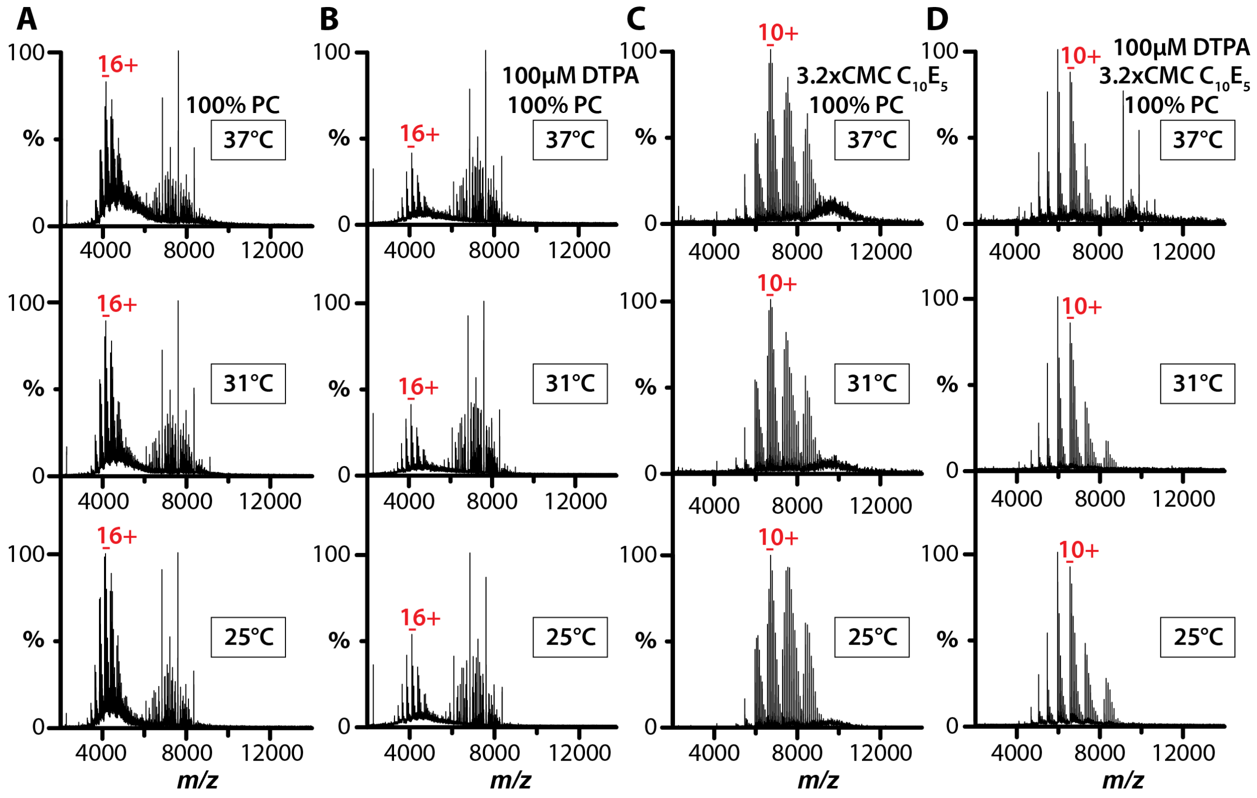

Figure S12. TRAAK in proteoliposomes containing 100% PC recorded at different temperatures. Native mass spectra of TRAAK consisting of A) 100% PC, B) 100% PC with 100µM DTPA, C) 100% PC with 3.2xCMC $C_{10}E_5$, and D) 100% PC with 100µM DTPA, and 3.2xCMC $C_{10}E_5$.

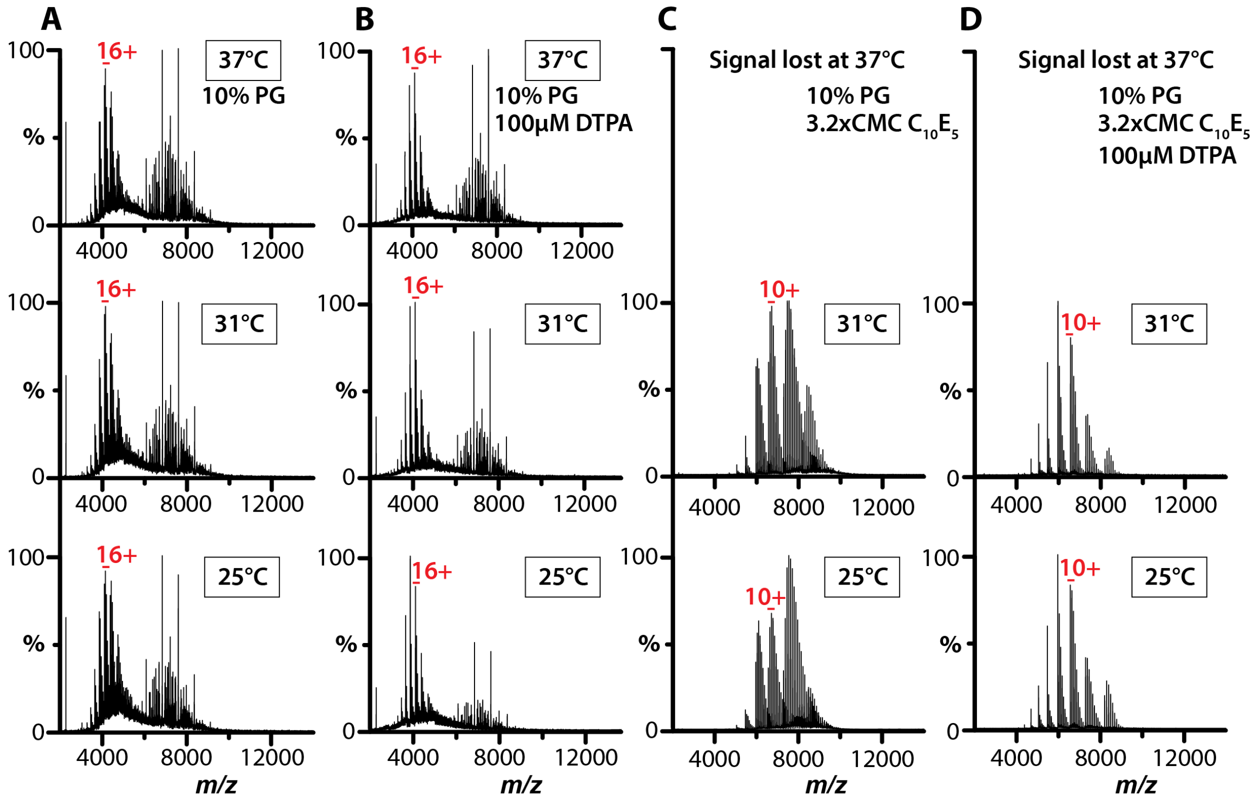

Figure S13. TRAAK in proteoliposomes containing PG recorded at different temperatures. Native mass spectra of TRAAK consisting of A) 10% PG, B) 10% PG with 100μM DTPA, C) 10% PG with 3.2xCMC $C_{10}E_5$, and D) 10% PG with 100μM DTPA, and 3.2xCMC $C_{10}E_5$.

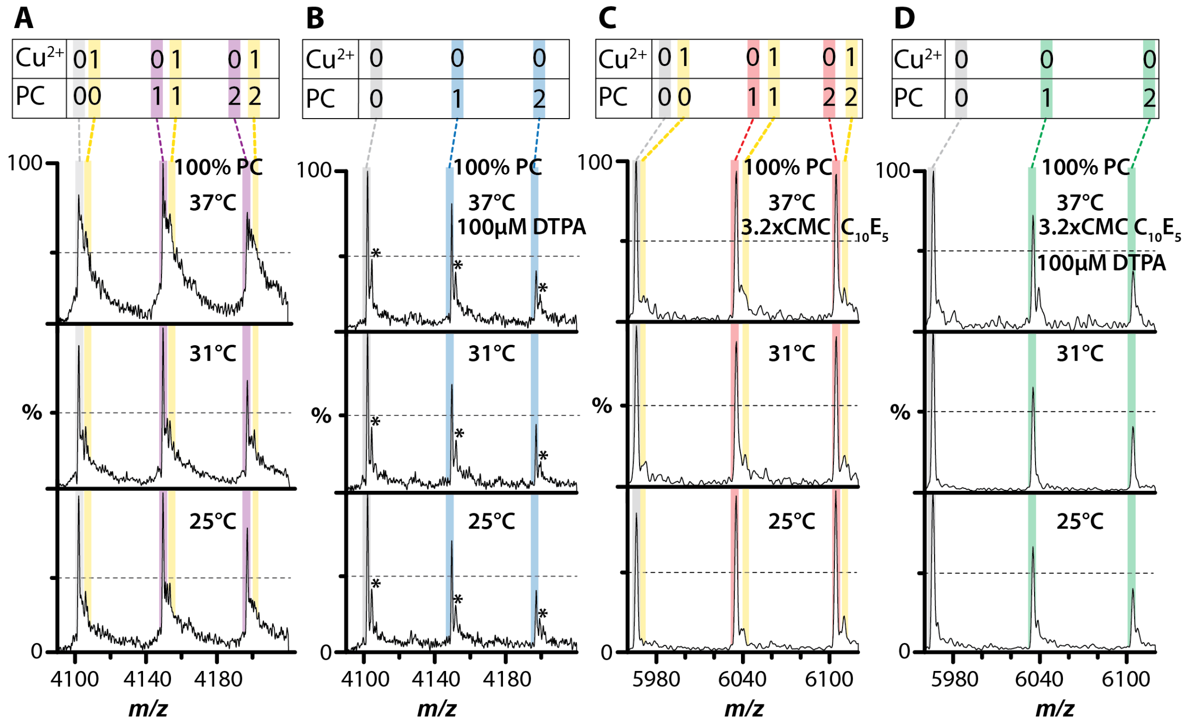

Figure S14. TRAAK in proteoliposomes recorded at different temperatures. Zoom of a charge state of the mass spectra of TRAAK in proteoliposomes consisting of A) 100% PC, B) 100% PC with 100μM DTPA, C) 100% PC with 3.2xCMC $C_{10}E_5$, and D) 100% PC with 100μM DTPA, and 3.2xCMC $C_{10}E_5$. For intact proteoliposomes with TRAAK, charge state 16+ was zoomed, whereas for detergent-solubilized proteoliposomes, charge state 11+ was zoomed.

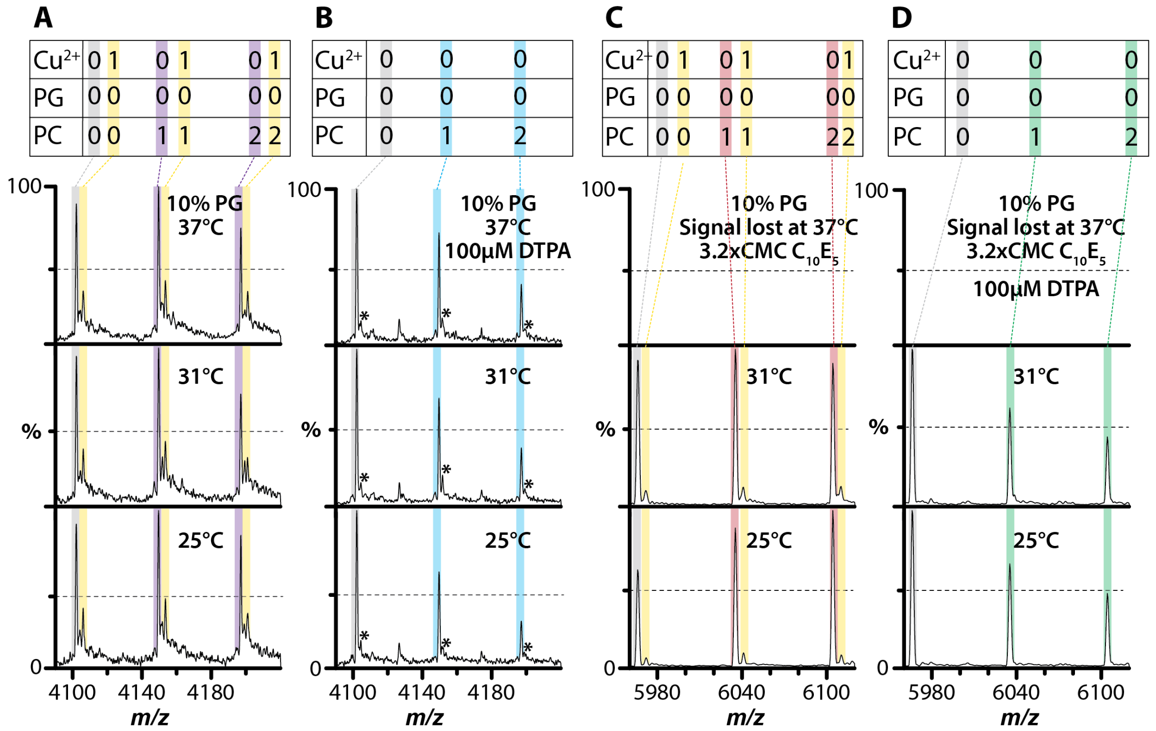

Figure S15. TRAAK in proteoliposomes containing 10% PG recorded at different temperatures. Zoom of a charge state of the mass spectra of TRAAK in proteoliposomes consisting of A) 10% PG, B) 100% PG with 100µM DTPA, C) 10% PG with 3.2xCMC $C_{10}E_5$, and D) 10% PG with 100µM DTPA, and 3.2xCMC $C_{10}E_5$. For intact proteoliposomes with TRAAK, charge state 16+ was zoomed, whereas for detergent-solubilized proteoliposomes, charge state 11+ was zoomed.

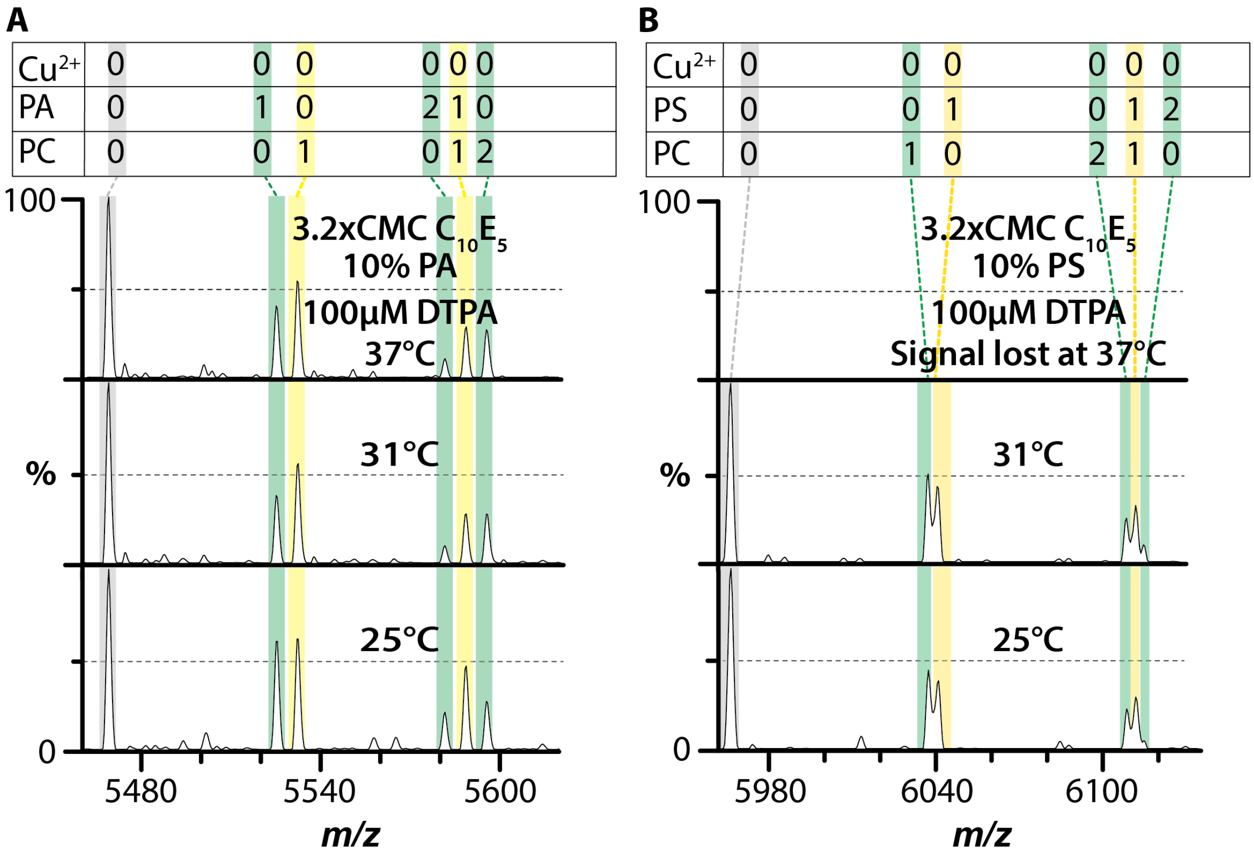

Figure S16. TRAAK in proteoliposomes of different compositions with 100µM DTPA and 3.2xCMC $C_{10}E_5$ recorded at different temperatures. Zoom of 11+ charge state of the mass spectra of TRAAK in proteoliposomes consisting of A) 10% PA and B) 10% PS.

Table S1. Instrument settings for analysis of AmtB proteoliposomes.

Parameters	***Values***
m/z range	2000-16000
Resolution	17500
SID	60
HCD	50
Spray Voltage (kV)	1.20
Capillary Temperature (°C)	250
Trapping Pressure	6.0
Source DC Offset (V)	30
Injection Flatpole DC (V)	11
Inter Flatpole Lens (V)	7.0
Bent Flatpole DC (V)	9
Transfer Multipole DC (V)	8
C-Trap Entrance Lens Tune Offset (V)	5

Table S2. Instrument settings for analysis of AmtB in detergent-solubilized proteoliposomes.

Parameters	***Values***
m/z range	2000-20000
Resolution	17500
SID	100
HCD	0
Spray Voltage (kV)	1.60
Capillary Temperature (°C)	250
Trapping Pressure	5.0
Source DC Offset (V)	60
Injection Flatpole DC (V)	4
Inter Flatpole Lens (V)	-20
Bent Flatpole DC (V)	10
Transfer Multipole DC (V)	6
C-Trap Entrance Lens Tune Offset (V)	5

Table S3. Instrument settings for analysis of TRAAK in proteoliposomes.

Parameters	***Values***
m/z range	2000-16000
Resolution	35000
SID	30-100
HCD	70
Spray Voltage (kV)	1.00-1.30
Capillary Temperature (°C)	200-250
Trapping Pressure	6.0
Source DC Offset (V)	30
Injection Flatpole DC (V)	12
Inter Flatpole Lens (V)	9
Bent Flatpole DC (V)	4
Transfer Multipole DC (V)	4
C-Trap Entrance Lens Tune Offset (V)	3

Table S4. Instrument settings for analysis of TRAAK in detergent-solubilized proteoliposomes.

Parameters	***Values***
m/z range	2000-20000
Resolution	17500
SID	65
HCD	100
Spray Voltage (kV)	1.50
Capillary Temperature (°C)	300
Trapping Pressure	7.0
Source DC Offset (V)	30
Injection Flatpole DC (V)	4
Inter Flatpole Lens (V)	-20
Bent Flatpole DC (V)	10
Transfer Multipole DC (V)	6
C-Trap Entrance Lens Tune Offset (V)	5

Supporting References

1. S. Kumar, L. Stover, L. Wang, H. Bahramimoghaddam, M. Zhou, D. H. Russell and A. Laganowsky, *Anal Chem*, 2024, **96**, 16768-16776.
2. S. Kumar, Y. Zhu, L. Stover and A. Laganowsky, *Anal Chem*, 2022, **94**, 13906-13912.
3. Y. Zhu, S. D. Yun, T. Zhang, J. Y. Chang, L. Stover and A. Laganowsky, *Chem Sci*, 2023, **14**, 14243-14255.
4. J. E. Keener, D. E. Zambrano, G. Zhang, C. K. Zak, D. J. Reid, B. S. Deodhar, J. E. Pemberton, J. S. Prell and M. T. Marty, *J Am Chem Soc*, 2019, **141**, 1054-1061.
5. J. W. McCabe, M. Shirzadeh, T. E. Walker, C. W. Lin, B. J. Jones, V. H. Wysocki, D. P. Barondeau, D. E. Clemmer, A. Laganowsky and D. H. Russell, *Anal Chem*, 2021, **93**, 6924-6931.
6. A. Laganowsky, E. Reading, J. T. Hopper and C. V. Robinson, *Nat Protoc*, 2013, **8**, 639-651.
7. M. T. Marty, A. J. Baldwin, E. G. Marklund, G. K. Hochberg, J. L. Benesch and C. V. Robinson, *Anal Chem*, 2015, **87**, 4370-4376.
